# Amplification-free RNA detection with CRISPR–Cas13

**DOI:** 10.1038/s42003-021-02001-8

**Published:** 2021-04-19

**Authors:** Hajime Shinoda, Yuya Taguchi, Ryoya Nakagawa, Asami Makino, Sae Okazaki, Masahiro Nakano, Yukiko Muramoto, Chiharu Takahashi, Ikuko Takahashi, Jun Ando, Takeshi Noda, Osamu Nureki, Hiroshi Nishimasu, Rikiya Watanabe

**Affiliations:** 1 Molecular Physiology Laboratory, Cluster for Pioneering Research, RIKEN, Saitama, Japan; 2 Department of Biological Sciences, Graduate School of Science, The University of Tokyo, Bunkyo-Ku, Tokyo Japan; 3 Structural Biology Division, Research Center for Advanced Science and Technology, The University of Tokyo, Meguro-Ku, Tokyo Japan; 4 Institute for Frontier Life and Medical Sciences, Kyoto University, Sakyo-Ku, Kyoto Japan

**Keywords:** Nanobiotechnology, Enzymes

## Abstract

CRISPR-based nucleic-acid detection is an emerging technology for molecular diagnostics. However, these methods generally require several hours and could cause amplification errors, due to the pre-amplification of target nucleic acids to enhance the detection sensitivity. Here, we developed a platform that allows “CRISPR-based amplification-free digital RNA detection (SATORI)”, by combining CRISPR-Cas13-based RNA detection and microchamber-array technologies. SATORI detected single-stranded RNA targets with maximal sensitivity of ~10 fM in <5 min, with high specificity. Furthermore, the simultaneous use of multiple different guide RNAs enhanced the sensitivity, thereby enabling the detection of the SARS-CoV-2 N-gene RNA at ~5 fM levels. Therefore, we hope SATORI will serve as a powerful class of accurate and rapid diagnostics.

## Abstract (web summary)

The authors develop a platform (SATORI) that enables accurate and rapid detection of single-stranded RNA at a single-molecule level without a pre-amplification step. As a proof-of-concept, they demonstrate its utility in detecting the SARS-CoV-2 N gene at a minimum concentration of ~5 fM, which is much lower than other amplification-free CRISPR-Cas-based methods.

## Introduction

Accurate and rapid nucleic-acid detection methods can contribute to early cancer diagnostics and virus pandemic prevention^1,2^. Currently, the demand is urgently increasing, since the novel coronavirus SARS-CoV-2 has caused over 117 million infections and 2.6 million deaths world wide (as of 8th March 2021)^3,4^. While reverse transcription quantitative polymerase chain reaction (RT-qPCR) is widely used as a “gold standard” method, CRISPR-based nucleic-acid detection, such as SHERLOCK and DETECTR, have recently been attracting keen attention as rapid and sensitive methods^4–10^. These CRISPR-based methods comprise a pre-amplification process of target nucleic acids and a subsequent detection mediated by CRISPR–Cas enzymes, such as Cas12a or Cas13a, via fluorescent or colorimetric readout. The pre-amplification process is necessary to increase the detection sensitivity, since the CRISPR-based methods lacking pre-amplification require ~0.5 h to detect picomolar amounts of a single-stranded RNA (ssRNA) target (the analytic limit of detection (LOD) is above 50 pM)^8,11^. However, the pre-amplification process increases the time to detection (by at least several tens of minutes), and could cause false-negative or -positive results due to amplification errors^12,13^.

To overcome these challenges, we combined the CRISPR–Cas13-based nucleic-acid detection system^9^ and our microchamber technology^14,15^, to develop a platform that enables accurate and rapid detection of ssRNA at a single-molecule level, termed SATORI (CRISPR-based amplification-free digital RNA detection). SATORI enabled rapid and sensitive detection of the N-gene RNA and whole genomic RNA from SARS-CoV-2, thereby highlighting the potential of SATORI as a powerful new class of rapid and robust viral diagnostics.

## Results

### Single-molecule detection of ssRNA by SATORI

**Fig. 1:**
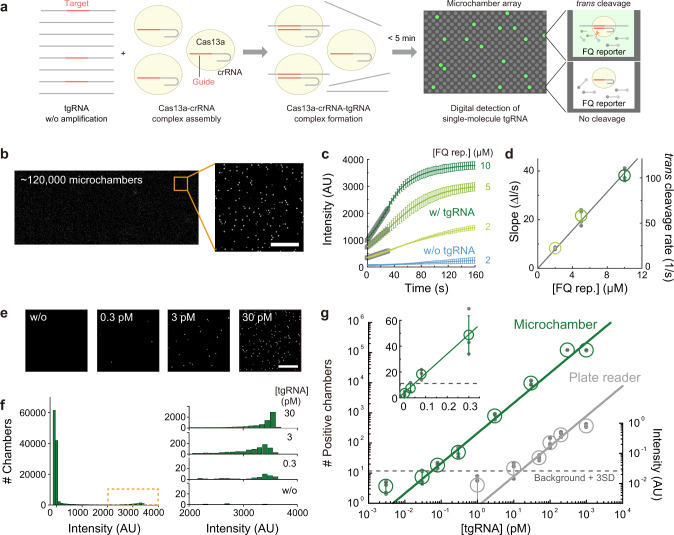
Amplification-free digital RNA counting with CRISPR–Cas13 in a microchamber array device. **a** Schematic illustration of SATORI. LwaCas13a–crRNA–tgRNA cleaves FQ reporters, leading to fluorescence increases in a microchamber array device. **b** Representative fluorescence image obtained by SATORI in the presence of 30 pM tgRNA. Forty images acquired by tiling imaging were combined. An enlarged view of the orange square is shown on the right. Scale bar is 50 μm. **c**, **d** Time course of fluorescence increase (**c**) and rates of *trans* cleavage (**d**) by LwaCas13a–crRNA–tgRNA with different concentrations of the FQ reporter. In **c**, average values of ten representative traces are shown with error bars (S.D.). In **d**, data are mean ± S.D. (*n* = 3 technical replicates). **e** Representative fluorescence images obtained with different concentrations of tgRNA. Scale bar is 50 μm. **f** Histograms of mean intensity values in each chamber (~120,000 chambers in the combined image). Enlarged views of the orange dotted box, with different concentrations of tgRNA, are shown on the right. **g** Comparison of LwaCas13a-mediated RNA detection methods in a microchamber device (SATORI, green) and a plate reader (gray), without recombinase polymerase amplification. Data are mean ± S.D. (*n* = 3 technical replicates), fitted to linear regressions. The dotted line is the value of the background mean + 3 S.D. LOD values for SATORI and the plate reader-based bulk assay were 56 fM and 11 pM, respectively.

We developed a platform “SATORI” by combining Cas13a-mediated RNA detection^9^ and our previously developed microchamber array device^14^. The device contains more than 1 × 10^6^ through-hole, femtoliter microchambers (*V* ~3 fL, *ϕ* = 2.5 μm, *h* = 0.6 μm), and thus enables massive and parallel observations of chemical reactions at single-molecule levels (Supplementary Fig. 1). Indeed, our microchamber device facilitated the development of highly sensitive and quantitative bioassays, such as digital enzyme-linked immunosorbent assay^16^ and single-molecule analysis of membrane proteins^14^. As a proof-of-concept experiment, we sought to detect a target ssRNA (tgRNA) in our microchamber device, using *Leptotrichia wadei* Cas13a (LwaCas13a) and the CRISPR RNA (crRNA), which were used in SHERLOCK^9^ (Fig. 1a). We pre-assembled the purified LwaCas13a protein with the crRNA (crRNA1) complementary to a 120-nt tgRNA (tgRNA1), and then added the LwaCas13a–crRNA1 complex into a sample solution containing the tgRNA1 and fluorophore quencher (FQ)-labeled RNA reporters (Supplementary Tables 1 and 2). We loaded the assay mixture into the microchamber device, and then observed fluorescence derived from the LwaCas13a-mediated FQ reporter cleavage, using a fluorescent microscope (Fig. 1a, Supplementary Fig. 2). After sealing the device, the fluorescence intensity significantly increased throughout the array (Fig. 1b, c), indicating that the LwaCas13a–crRNA1 complexes recognized the tgRNA1 and cleaved the FQ reporters in *trans* in the microchambers. Real-time recording revealed that the fluorescence intensities in the chambers reached plateaus in 2 min, due to the small chamber volume (*V* ~3 fL) and the robust cleavage activity of LwaCas13a (*k* = 1.1 × 10^7^ × [FQ rep.] M^−1^ s^−1^) (Fig. 1c, d, and Supplementary Figs. 3 and 4). In SATORI, we obtained fluorescence images from ~1.2 × 10^5^ chambers within a few minutes, and therefore, it only requires <5 min in total, which is much shorter than the durations of other CRISPR-based methods^4,11^.

When the sample solution was diluted to ~1:1 ratio of tgRNA per chamber, the fluorescence intensity in the array was not homogeneously distributed (Fig. 1b). We defined chambers with mean intensity over 2000 (background + 20 S.D.) as positive chambers containing an LwaCas13a–crRNA1–tgRNA1 complex. The number of positive chambers linearly increased, depending on the tgRNA concentrations (30 fM to 300 pM) (Fig. 1e–g). These results suggested that each chamber stochastically contained one or zero LwaCas13a–crRNA1–tgRNA1 complex under these conditions, and the fluorescence in each positive chamber was derived from the reporter cleavage by the single LwaCas13a–crRNA1–tgRNA1 molecule. Notably, SATORI detected tgRNA1 with an LOD of 56 fM (“Materials and methods”), which is far below the detection limit of a plate reader-based bulk assay (Fig. 1g, Supplementary Fig. 5), demonstrating the advantage of digital detection in a microchamber array.

**Fig. 2:**
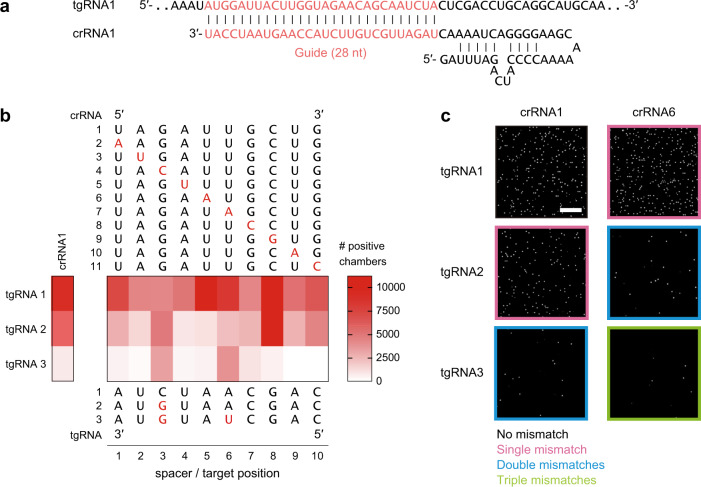
Sequence specificity of SATORI. **a** Sequences of the crRNA and tgRNA. **b** Effects of single, double, and triple mismatches on the number of positive chambers. Mutations are highlighted in red. Data are means (*n* = 3 technical replicates). **c** Representative fluorescence images obtained with different combinations of the crRNA and tgRNA sequences.

To investigate the specificity of SATORI, we examined the effects of mismatches between crRNA and tgRNA on the number of positive chambers, using 11 crRNAs (crRNA1–11) and three tgRNAs (tgRNA1–3), which were used in a previous study^9^ (Fig. 2a). Single mismatches did not substantially affect the number of positive chambers (Fig. 2b). In contrast, double and triple mismatches reduced them by ~5- and ~25-fold, respectively (Fig. 2b), while they did not affect the fluorescence kinetics (Supplementary Fig. 6). These results indicated that the mismatches reduced the number of active LwaCas13a–crRNA–tgRNA molecules, as previously suggested^17^, but did not affect the *trans* cleavage activity of LwaCas13a. The effects were dependent on the mismatch positions, and double mismatches at position 3 and position 2, 4, or 5 had the pronounced effects (Fig. 2b, c), consistent with a previous study with SHERLOCK^9^, confirming the validity of our experiments.

### Rapid and sensitive detection of SARS-CoV-2 by SATORI

**Fig. 3:**
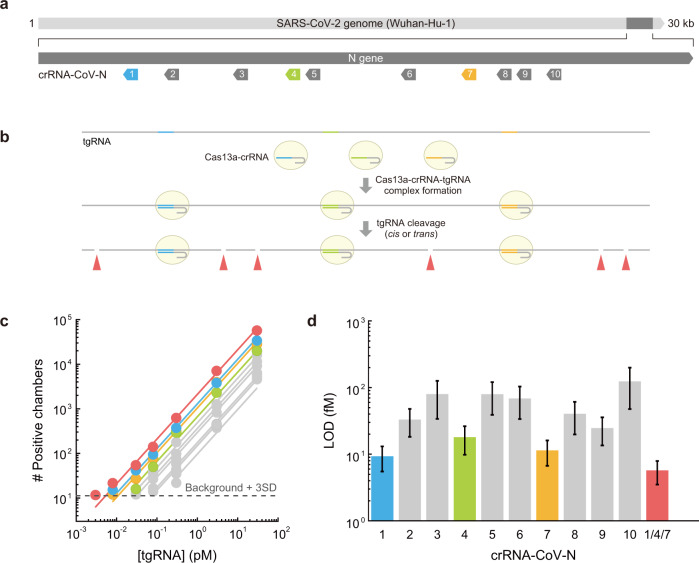
SARS-CoV-2 detection by SATORI. **a** Schematics of the crRNAs targeting different sites in the SARS-CoV-2 N-gene (Wuhan-Hu-1). **b** Schematics of multiplexed SATORI. **c** Numbers of positive chambers obtained with the crRNAs (crRNA-CoV-N-1–10) at different concentrations of tgRNA (SARS-CoV-2 N-gene). Data with crRNA-CoV-N-1, -4, and -7 are colored blue, yellow-green, and orange, respectively, while those with a combination of these crRNAs are highlighted in red. Solid lines represent linear regressions. **d** LOD values of the SARS-CoV-2 N-gene RNA for different crRNAs.

To examine the ability of SATORI to detect SARS-CoV-2, we performed SATORI using 10 crRNAs (crRNA-CoV-N-1–10) targeting different regions of the SARS-CoV-2 N-gene RNA (~1 kb), which is also targeted in the US CDC RT-qPCR assays^18^ (Fig. 3a, Supplementary Fig. 7). Notably, SATORI detected the SARS-CoV-2 N-gene RNA with LOD values of 9.3–123 fM (Fig. 3c, d). These results indicated that the efficiency of SATORI varies depending on the crRNA guide sequences, as also observed in SHERLOCK^9^. Upon target RNA binding, LwaCas13a–crRNA complexes cleave RNA targets in *cis* or *trans* into multiple RNA fragments. Thus, the simultaneous use of multiple crRNAs complementary to different regions of a target RNA could generate multiple LwaCas13a–crRNA–tgRNA molecules from a single target RNA molecule, thereby increasing the potential number of positive chambers (Fig. 3b). To test this hypothesis, we simultaneously used three crRNAs (crRNA-CoV-N-1, -4, and -7), which exhibited the highest sensitivity among the tested crRNAs (the LOD values for N-1, -4, and -7 were 9.3 ± 3.8, 18.0 ± 8.2, and 11.4 ± 4.7 fM, respectively). Indeed, the simultaneous use of these three crRNAs resulted in the LOD value of 5.7 ± 2.2 fM (3.4 × 10^6^ copies/mL) (Fig. 3c, d), consistent with the theoretical value (4.0 ± 1.0 fM) (“Materials and methods”).

**Fig. 4:**
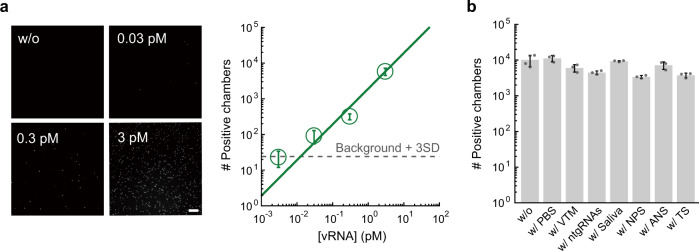
Practicability of SATORI for clinical applications. **a** Digital detection of RNA extracted from SARS-CoV-2 virus (vRNA). The representative fluorescence images and the number of positive chambers, obtained with the crRNA-CoV-N1 at different concentrations of vRNA, are shown. **b** Effects of contaminants on SATORI. SATORI assays were performed with the crRNA1 and the tgRNA1 in the presence of 10% PBS, 70% virus transport medium (VTM), 3 ng/μL nontarget RNAs (ntgRNAs), 10% saliva, nasopharyngeal swab (NPS), anterior nasal swab (ANS), or throat swab (TS).

To examine the applicability of SATORI to clinical samples, we performed SATORI using the whole genomic RNA (~30 kb) of SARS-CoV-2 isolated from the cruise ship “Diamond Princess”, the first large outbreak cluster in Japan. We found that SATORI with the crRNA-CoV-N-1 detects the SARS-CoV-2 genomic RNA with LOD of 12.8 fM (Fig. 4a), which is comparable to LOD for the N-gene RNA fragment (9.3 fM). We also examined whether SATORI is affected by contaminants, such as virus transport media, saliva, nasopharyngeal swabs, anterior nasal swabs, throat swabs, and nontarget RNAs, which are abundant in clinical specimens and affect conventional qPCR and other CRISPR-based methods^7,19^. We observed almost the same number of positive chambers in the presence of these contaminants (Fig. 4b), suggesting the compatibility of SATORI to raw clinical specimens. Together, these results indicated the potential of SATORI for SARS-CoV-2 diagnostics.

## Discussion

In this study, we demonstrated that SATORI is an accurate, rapid, and robust ssRNA detection platform, and it has several technical advantages as compared to other ssRNA detection methods. First, unlike conventional PCR-based methods, SATORI is not affected by amplification errors, since it counts the number of target RNA molecules at a single-molecule level. Second, SATORI requires less time (<5 min) for detection as compared to other methods (>0.5 h^11^). Third, SATORI is more robust against contaminants such as saliva, suggesting the potential of SATORI for the direct application to clinical samples without an RNA purification process. In addition, SATORI is tolerant to single mismatches between the guide and target sequences, as reported for other CRISPR-based methods, e.g., SHERLOCK^9^. This mismatch tolerance may be advantageous for the detection of ssRNA viruses, which could acquire point mutations^20^. Thus, SATORI could be used for a more rapid and robust primary screening for infections of ssRNA viruses, e.g., SARS-CoV-2, HIV, Zika, Ebola, and Influenza, as combined with the current antigen test, before a more accurate but time-consuming qPCR test. Furthermore, SATORI combined with Cas12a would enable amplification-free double-stranded DNA detection, which could be applied to the diagnosis of DNA virus infections and the detection of circulating tumor DNAs for cancer diagnostics.

The sensitivity of SATORI (LOD of 5.7 fM (3.4 × 10^6^ copies/mL)) was ~10^4^- and 3-times higher than those of other amplification-free CRISPR methods (~50 pM (~3 × 10^10^ copies/mL))^11^ and antigen tests (~17 fM (1.0 × 10^7^ copies/mL))^21^, respectively. These observations indicated that SATORI should be sensitive enough to detect SARS-CoV-2 RNA in clinical specimens from most patients (~10^6^–10^9^ copies/mL on average)^22^, although SATORI is ~10^3^-times less sensitive than other amplification-based methods, such as SHERLOCK (~2 aM (~10^3^ copies/mL))^9,11^, DETECTR (~20 aM (~10^4^ copies/mL))^7^, and qPCR (~2–20 aM (~10^3^–10^4^ copies/mL))^23^.

During the revision process of this paper, Fozouni et al.^24^ reported a CRISPR–Cas13a-based amplification-free RNA detection method. Consistent with our findings, they showed that combinations of multiple crRNAs improved the sensitivity of Cas13a-mediated RNA detection, and detected SARS-CoV-2 RNA in clinical specimens from infected patients^24^. However, there are notable differences between their method and SATORI. Fozouni et al.^24^ used *Leptotrichia buccalis* Cas13a (LbuCas13a) and performed fluorescence measurements in 384-well plates. In contrast, we used LwaCas13a and detected fluorescent signals in a microchamber device at the single-molecule level. It will be interesting to examine whether the sensitivity of SATORI can be further improved by the use of LbuCas13a rather than LwaCas13a and the simultaneous use of a greater number of more effective crRNAs. In addition, assay sensitivity may be increased by optimizing the sample concentration step, as reported for other methods^25^.

SATORI will be practical in clinical laboratories in terms of cost and scalability. SATORI currently costs approximately US$9.10 (US$5.20 for the fabrication of one microchamber and US$3.90 for reagents) (Supplementary Tables 5 and 6), which is comparable to qPCR and antigen tests (US$1.21–4.39)^26^. Vogels et al.^26^ developed SalivaDirect, an inexpensive saliva-based nucleic acid extraction-free qPCR method, and demonstrated that saliva is a valid specimen for SARS-CoV-2 diagnosis. Given that SATORI is not inhibited by the presence of saliva, it has potential for further cost reduction. In addition, the costs of SATORI could be reduced by mass production of the microchamber devices. We conducted SATORI assays using a wide-field normal fluorescent microscope equipped with a 20× objective lens, and analyzed images using “ImageJ software”, a freely available software tool (Fig. 4). Therefore, it is relatively easy to implement SATORI using compact and portable fluorescent microscopes for general use, as previously reported^27^. These features support the future scalability and practicability of SATORI in clinical laboratories. Given our findings reported here, we believe that SATORI will be a key technology for future diagnostics.

## Methods

### Fabrication of microchamber array devices

Hydrophobic through-hole structures were fabricated on a thin glass substrate by conventional photolithography^16^. A 32 mm × 24 mm cover glass (No. 1, Matsunami) was incubated overnight and sonicated for 1 h in an 8 N KOH solution, rinsed with pure water, and dried using an air blow gun. Perfluoro-polymer (9% CYTOP, AGC) was spin-coated on the glass at 1000 rpm or 4000 rpm for 30 s, and then baked at 80 °C for 10 min and 180 °C for 1 h. The thickness of the CYTOP layer (1.6 μm or 0.6 μm) was determined with a laser microscope (VK-X1100, Keyence). Positive photoresist (AZ P4620, AZ Electronic Materials) was spin-coated on the CYTOP layer at 7500 rpm for 30 s, and then cured at 100 °C for 5 min. After rehydration of the photoresist at 25 °C for 5 min under 60% humidity, the glass was exposed to UV light, using a mask aligner (PEM-800, Union) and a chrome photomask with 1 μm holes, and then incubated for 1.5 min in developer (AZ300 MIF, AZ Electronic Materials). The photoresist-uncovered CYTOP was removed by dry etching with O_2_ plasma (DES-101E, YAC). The fabrication of the microchamber devices was completed by the removal of the remaining photoresist by sequential rinses with acetone, 2-propanol, and pure water. The quality of the devices was evaluated with the aforementioned laser microscope. Devices with hole-diameters of 2.5 ± 0.2 μm were selected and used for our experiments (Supplementary Fig. 1). A flow cell was constructed on the device, by attaching a U-shaped frame chamber seal (SLF0601, Bio-Rad) and a custom-made glass block with an inlet port (Tsubaki Glass Kogyosyo) (Supplementary Fig. 1).

### Protein preparation

*Escherichia coli* Rosetta 2(DE3) was transformed with the pET-LwaCas13a plasmid, and the cells were cultured at 37 °C in 100 mL TB medium, supplemented with chloramphenicol and kanamycin. When the OD_600_ reached 0.6–0.8, the bacterial culture was cooled on ice for 10 min, and then further cultured at 20 °C for 20 h with 0.1 mM IPTG. The *E. coli* cells were collected, suspended in 5 mL buffer A (20 mM Tris-HCl (pH 8.0), 1 M NaCl, and 20 mM imidazole), and disrupted by a sonicator (Q500, QSONICA). After centrifugation at 15,000 rpm for 15 min, the supernatant was loaded onto a Ni-Sepharose High Performance column (GE Healthcare), equilibrated with buffer A. The protein was eluted with buffer B (20 mM Tris-HCl (pH 8.0), 300 mM NaCl, and 400 mM imidazole). The protein was loaded onto a HiTrap Heparin HP column (1 mL; GE Healthcare), equilibrated with buffer C (20 mM Tris-HCl (pH 8.0) and 300 mM NaCl). The protein was eluted with a linear gradient of 0.3–2 M NaCl. The fractions were analyzed by SDS-PAGE, and peak fractions were pooled. The protein concentration was determined according to the *A*_280_ value measured by a NanoDrop spectrophotometer (Thermo Scientific). Aliquots of the purified LwaCas13a protein were quickly frozen in liquid nitrogen and stored at −80 °C until measurements.

### RNA preparation

The crRNA or tgRNA (Supplementary Tables 1 and 2) was transcribed in vitro with T7 RNA polymerase at 37 °C for 1 h, using a partially double-stranded DNA template (Supplementary Table 4). To remove the double-stranded RNA contaminations, as reported previously^28^, the transcribed RNA was incubated with RNaseIII (New England Biolabs) at 37 °C for 30 min, and then purified by 8% native polyacrylamide gel electrophoresis. The RNA concentration was determined from the *A*_260_ value measured by the NanoDrop spectrophotometer.

SARS-CoV-2 (SARS-CoV-2/Hu/DP/Kng/19-027) was grown in VeroE6/TMPRSS2 cells (JCRB 1819), which was maintained in Dulbecco’s modified Eagle’s medium (DMEM, Sigma-Aldrich) containing 5% fetal calf serum (FCS) and 1% penicillin/streptomycin. The whole genomic RNA of SARS-CoV-2 was extracted using RNeasy Mini kit (Qiagen) and stored at −80 °C until use.

### SATORI

The purified LwaCas13a protein was diluted to 1 μM with buffer D (20 mM HEPES (pH 7.5), 150 mM KCl, 10 mM MgCl_2_, and 0.5 mM DTT). LwaCas13a (1 μM) was mixed with an equal volume of the crRNA (625 nM) in nuclease-free water, and then incubated at 37 °C for 10 min. The LwaCas13a–crRNA complex (4 μL) was then added to an assay mixture (46 μL): buffer D containing 10 μM FQ reporter (Integrated DNA Technologies, Supplementary Table 3), 500 μM Triton X-100 (Nacalai Tesque), 20 μM Alexa Fluor™ 647 C_2_ Maleimide (Thermo Scientific), and various amounts of tgRNA. The mixture (50 μL) was loaded onto the microchamber device from an inlet port on the glass block, and then incubated at 25 °C for a few minutes. The device was set on a motorized XY scanning stage of a confocal microscope (A1, Nikon), equipped with a 60× oil-immersion lens (NA = 1.40, Apo, Lambda S) and 488 and 640 nm lasers. For assays with the whole genomic RNA of SARS-CoV-2, the device was set on a motorized XY scanning stage of a wide-field microscope (IX71, Olympus), equipped with a 20× dry lens (NA = 0.45) and 488 and 640 nm lasers. Then, 40 μL hexadecane (296317, Sigma-Aldrich) was loaded into the device at a speed of 4 μL/s, using a customized electric pipettor (ICOMES). Fifty seconds after the loading, fluorescence tiling imaging for 40 stage positions was started, and imaging was conducted at 25 °C.

To examine the effects of contamination on SATORI, PBS (10%), virus transport medium (VTM) (70%, SGI), and standard RNA (3 ng/μL, Qubit RNA BR Standard #2, Thermo Scientific) was added to the assay mixture, and the SATORI assays were conducted. The VTM was composed of cell-culture medium, bovine serum albumin, penicillin, streptomycin, gentamicin, and amphotericin B. For assays with 70% VTM, KCl was removed from the assay mixture.

For assays with saliva, 10% saliva was diluted in 20 mM HEPES (pH 7.5) containing 10 mM MgCl_2_, 20 U of RNase inhibitor (AM2694, Thermo Scientific), 100 mM DTT and 1 mM Triton-X100, and heated on a heat block (MyBL-10, As One) at 90 °C for 5 min. The various amounts of tgRNA, 5 μM FQ reporter, 20 μM Alexa Fluor™ 647 C_2_ Maleimide, and the LwaCas13a-crRNA complex were then added to the solution, and the SATORI assays were conducted as describe above.

Nasopharyngeal swab, anterior nasal swab, and throat swab samples were collected according to a Centers for Disease Control and Prevention (CDC) protocols. Nasopharyngeal swab and throat swab samples were prepared in 1 mL buffer D containing 100 mM DTT and 1 mM Triton X-100. For assays with anterior nasal swab samples, KCl was removed from the buffer D. After a heating at 90 °C for 5 min, 20 U of RNase inhibitor, tgRNA, 10 μM FQ reporter, 20 μM Alexa Fluor™ 647 C_2_ Maleimide, and the LwaCas13a–crRNA complex were added to the sample, and the SATORI assays were conducted.

For the multiplexed SATORI assay, LwaCas13a (4 μM) was mixed with an equal volume of 2.5 μM crRNA (crRNA-CoV-N-1, -4, or -7), and then incubated at 37 °C for 10 min. Each LwaCas13a–crRNA complex (1 μL) was added to the assay mixture (47 μL), and the SATORI assays were conducted.

### Kinetics measurement of *trans* cleavage activity

For the measurement of the *trans* cleavage activity of LwaCas13a at the single-molecule level, imaging was immediately started after the hexadecane loading onto the microchamber device. Fluorescence images on a single stage point were recorded at time intervals of 3 s. The number of cleaved FQ reporters was calculated based on the mean fluorescence intensity in each chamber, using a calibration curve of mean fluorescence intensity to FAM concentration (Supplementary Fig. 4), and the chamber volume was measured by a laser microscope (Keyence). To obtain the calibration curve, fluorescein-conjugated ssRNA without any quenchers (56-FAM/rUrUrUrUrU, Integrated DNA Technology) in buffer D, containing 500 μM Triton X-100 and 20 μM Alexa Fluor 647, were loaded into the microchamber device, followed by sealing with hexadecane, and the imaging was conducted using the same setups as in the SATORI assay.

Bulk *trans* cleavage assays were performed, using LwaCas13a (45 nM), crRNA (22.5 nM), FQ reporter (125 nM), murine RNase inhibitor (0.5 μL, New England Biolabs), and various amounts of tgRNA in assay buffer (20 mM HEPES (pH 6.8), 60 mM NaCl, and 6 mM MgCl_2_). Reactions were incubated at 37 °C for 10 min, and fluorescence was detected at every 1 min at excitation (470 nm) and emission (520 nm) wavelengths on a fluorescence microplate reader (SpectraMax ID3, Molecular Devices).

### Data analysis

Image processing, including spherical aberration correction, drift correction, background subtraction, and extraction of fluorescence intensity in chambers, was conducted using ImageJ/Fiji^29^. ImageJ plugin, Template Matching and Slice Alignment (https://sites.google.com/site/qingzongtseng/template-matching-ij-plugin#credit) were used for the drift correction. All of the processes were automated, using a macro program. A series of the extracted intensity data were analyzed, using a program written in Python, with Anaconda3 (https://www.anaconda.com/).

LOD values were defined as follows: Output values obtained with different concentrations of tgRNA were fitted to a linear curve (the output values correspond to the number of positive chambers and the fluorescence intensities in SATORI and the plate reader-based method, respectively). The means + 3 S.D. for output values obtained without tgRNA were measured, and the crossing point of the linear curve and the mean + 3 S.D. value was determined. The concentration corresponding to the crossing point was defined as the LOD value.

### Theoretical calculation of LOD for multiplexed SATORI

From the single-molecule measurements, LOD values were determined as the crossing points of the fitted linear regression lines and the background mean + 3 S.D. value, *B* (Figs. 1g and 3c). The number of positive chambers, *N*, was represented as a function of LOD.

1 $$N = \frac{B}{{\rm{LOD}}}[{\rm{tgRNA}}]$$

Upon binding target RNA, LwaCas13a–crRNA complexes cleaved RNA targets in *cis* or *trans* into multiple RNA fragments. Thus, in the presence of LwaCas13a with three different crRNAs, three LwaCas13a–crRNA–tgRNA molecules were generated from a single target RNA molecule. Accordingly, the theoretical *N*_*mp*_ for multiplexed SATORI with the combination of the three crRNA was a sum of *N*_*N*1_, *N*_*N*4_, and *N*_*N*7_ as follows:

2 $$\begin{array}{l}N_{mp} = N_{N1} + N_{N4} + N_{N7} = B\left( {\frac{1}{{\rm{LOD}}_{N1}} + \frac{1}{{\rm{LOD}}_{N4}} + \frac{1}{{\rm{LOD}}_{N7}}} \right)\left[ {\rm{tgRNA}} \right]\\ \quad \quad \quad \quad \quad \quad \quad \quad \quad \quad = \frac{B}{{\rm{LOD}}_{mp}}[{\rm{tgRNA}}]\end{array}$$

where LOD_N1_, LOD_N4_, LOD_N7_, and LOD_mp_, were LOD values for N-1, -4, -7, and their combination, respectively. By comparing the coefficients, LOD_mp_ was given as a function of LOD_N1_, LOD_N4_, and LOD_N7_ as follows.

3 $$\frac{1}{{\rm{LOD}}_{mp}} = \frac{1}{{\rm{LOD}}_{N1}} + \frac{1}{{\rm{LOD}}_{N4}} + \frac{1}{{\rm{LOD}}_{N7}}$$

Based on Eq. (3), the theoretical LOD_mp_ was calculated as 4.0 ± 1.0 fM, and was coincided with the experimental result, 5.7 ± 2.2 fM (Fig. 3c, d).

### Statistics and reproducibility

All the measurements, described in this paper, were taken from distinct samples, and all experiments performed on the paper were successfully replicated more than three times.

### Reporting summary

Further information on research design is available in the Nature Research Reporting Summary linked to this article.

## Supplementary information

Supplementary Information

Description of Additional Supplementary Files

Supplementary Data 1

Reporting Summary

## Data Availability

All source data used for generating graphs and charts in main figures are included in Supplementary Data 1. The data that support the findings of this study are available from the corresponding author upon reasonable request.
