# Supplementary material for: Amplification-free RNA detection with CRISPR–Cas13
Source: Commun Biol. 2021 Apr 19;4:476. doi: 10.1038/s42003-021-02001-8 (PMC8055673; doi:10.1038/s42003-021-02001-8)
Supplement: Supplementary file 1 — Supplementary Information [file 42003_2021_2001_MOESM1_ESM.pdf]

Supplementary Information

Amplification-free RNA detection with CRISPR-Cas13

Hajime Shinoda, Yuya Taguchi, Ryoya Nakagawa, Asami Makino, Sae Okazaki, Masahiro Nakano, Yukiko Muramoto, Chiharu Takahashi, Ikuko Takahashi, Jun Ando, Takeshi Noda Osamu Nureki, Hiroshi Nishimasu, Rikiya Watanabe

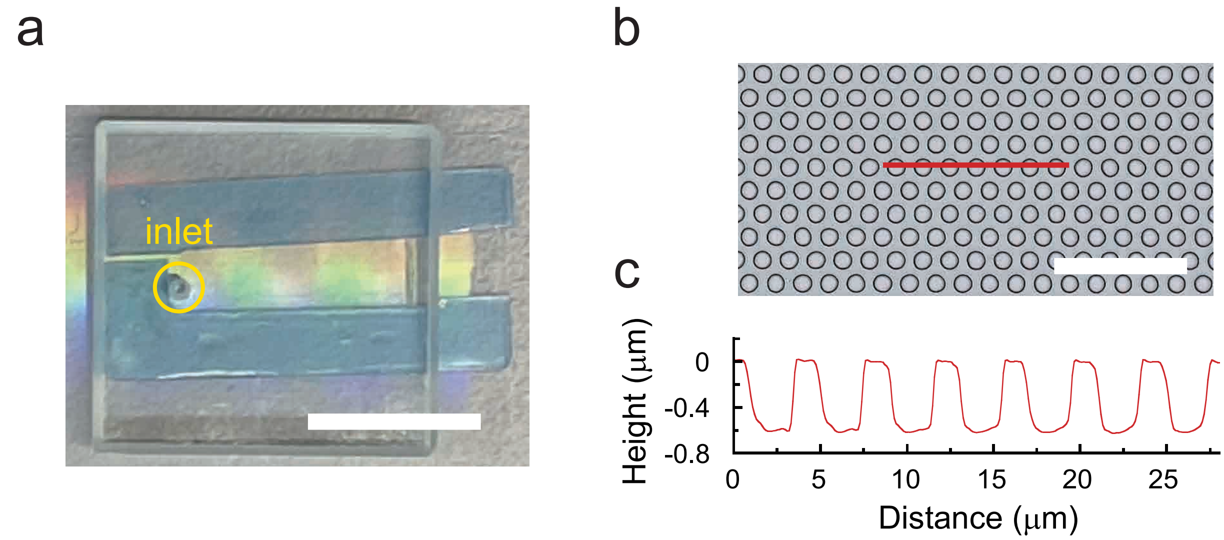

Supplementary Figure 1. Microchamber device

(a) Top view of the microchamber device. A glass block with an inlet port is attached to a glass substrate containing 1,000,000 microchambers, with a U-shaped spacer seal (light blue) in between. Scale bar, 10 mm. (b) Surface image of the microchamber on a glass substrate, acquired by 3D confocal laser microscopy. Scale bar, 20 μm. (c) Line profile of the cross section on the red line in (b). The average diameter, height, and volume of the chambers are 2.5 μm, 0.6 μm, and 2.9 fL, respectively.

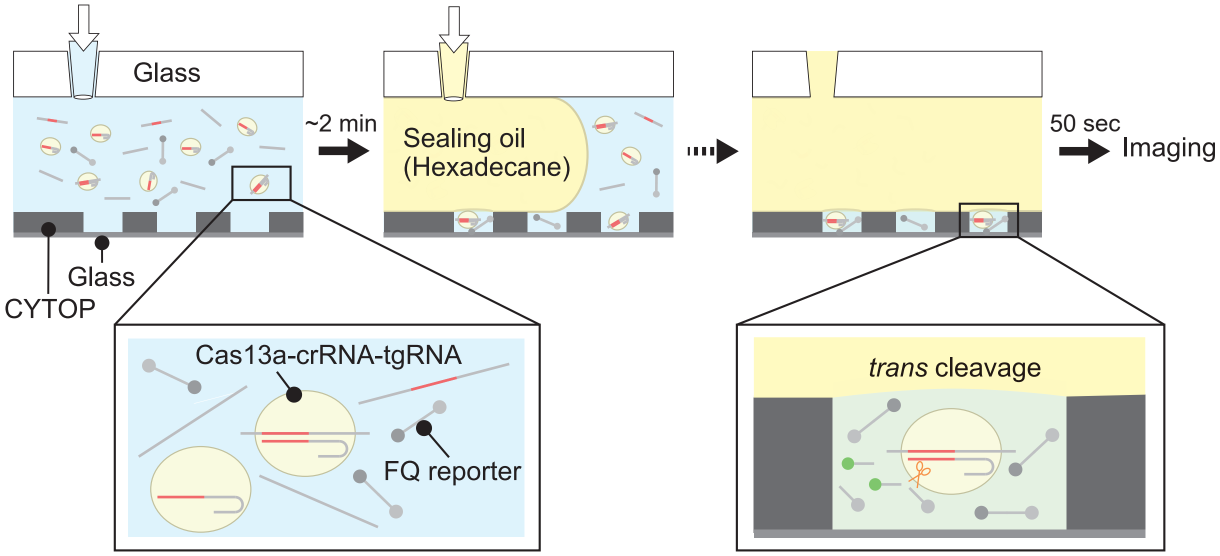

Supplementary Figure 2. Schematics of assay mixture encapsulation into microchambers

The assay mixture, containing LwaCas13a-crRNA, tgRNA, and FQ-reporter, is loaded into the microchamber device from the inlet in the top glass block. A few minutes afterwards, hexadecane is loaded from the inlet. Because the hydrophobic hexadecane has high affinity to the hydrophobic CYTOP layer on the glass substrate and less affinity to water, the assay mixture is exclusively trapped inside the chambers.

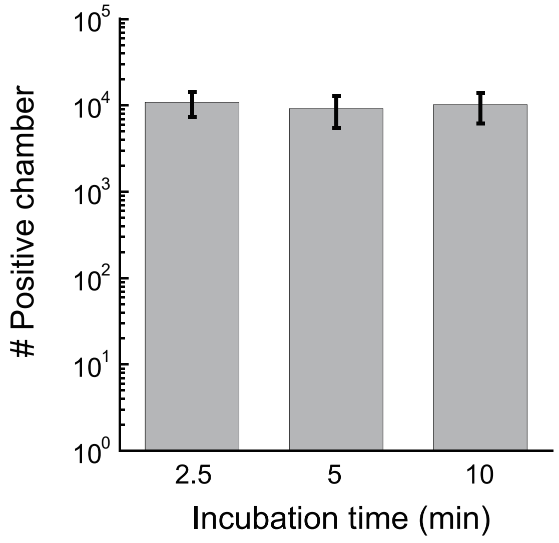

Supplementary Figure 3. Effects of incubation time with tgRNA

The LwaCas13a-crRNA binary complex was mixed with 30 pM tgRNA, loaded into the microchamber device, and then incubated at 25°C for the indicated times, followed by sealing with hexadecane. The numbers of positive chambers are shown. Data are mean ± S.D. (n = 3 technical replicates).

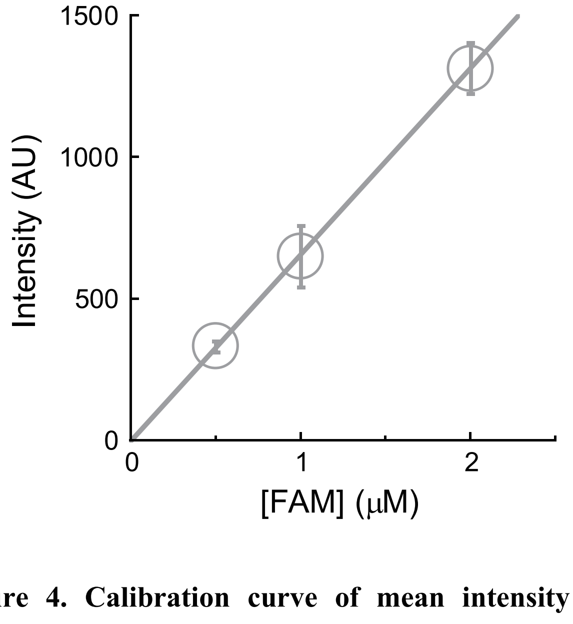

Supplementary Figure 4. Calibration curve of mean intensity in each chamber to FAM concentration

The FAM-conjugated RNA without any quenchers was loaded into the microchamber device, followed by sealing with hexadecane, and the fluorescence images were then collected. The solid line represents the linear regression, and data are mean ± S.D. (n = 3 technical replicates).

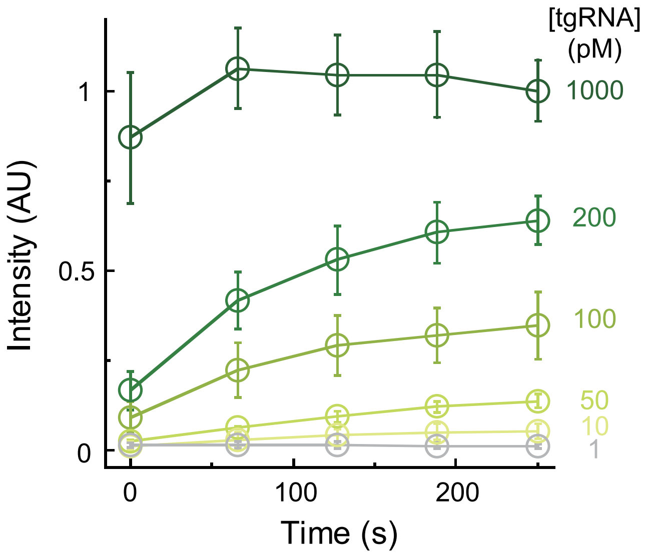

Supplementary Figure 5. Plate reader-based RNA detection with LwaCas13a

Time courses of fluorescence increases caused by the FQ reporter cleavage are shown. The assay mixture containing LwaCas13a-crRNA and the FQ reporters was mixed with a tgRNA-containing solution at the indicated concentrations, and then the fluorescence time-course was monitored. The fluorescence intensities were normalized by the values obtained with 1 nM tgRNA at 250 s incubation. Data are mean ± S.D. (n = 3 technical replicates).

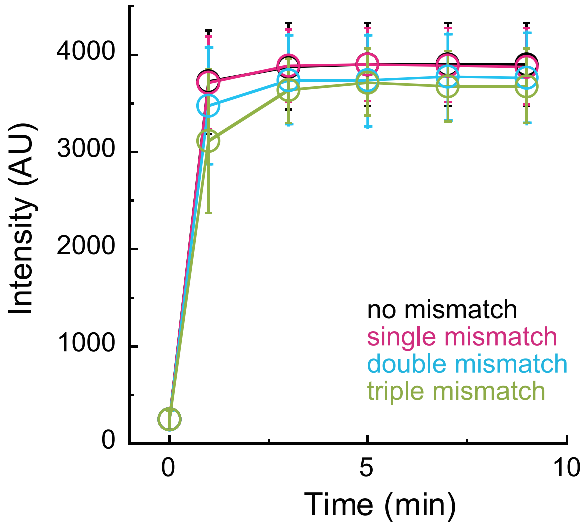

Supplementary Figure 6. Effects of mismatches between crRNA and tgRNA on the FQ reporter cleavage

Time courses of fluorescence increases caused by FQ reporter cleavage in the absence of mismatches (crRNA1 & tgRNA1, black) or in the presence of single (crRNA6 & tgRNA1, pink), double (crRNA6 & tgRNA2, blue), and triple mismatches (crRNA6 & tgRNA3, green). Data are mean ± S.D. (n = 3 technical replicates).

AUGUCUGAUAAUGGACCCCAAAAUCAGCGAAAUGCACCCCGCAUUACGUUUGGUGGACCCUCAGAUUCAACUGGC

AGUAACCAGAAUGGAGAACGCAGUGGGGCGCGAUCAAAACAACGUCGGCCCCAAGGUUUACCCAAUAAUACUGCG

UCUUGGUUCACCGCUCUCACUCAACAUGGCAAGGAAGACCUUAAAUUCCCUCGAGGACAAGGCGUUCCAAUUAAC

N-1

ACCAAUAGCAGUCCAGAUGACCAAAUUGGCUACUACCGAAGAGCUACCAGACGAAUUCGUGGUGGUGACGGUAAA

N-2

AUGAAAGAUCUCAGUCCAAGAUGGUAUUUCUACUACCUAGGAACUGGGCCAGAAGCUGGACUUCCCUAUGGUGCU

AACAAAGACGGCAUCAUAUGGGUUGCAACUGAGGGAGCCUUGAAUACACCAAAAGAUCACAUUGGCACCCGCAAU

N-3

CCUGCUAACAAUGCUGCAAUCGUGCUACAACUUCCUCAAGGAACAACAUUGCCAAAAGGCUUCUACGCAGAAGGG

N-4

AGCAGAGGCGGCAGUCAAGCCUCUUCUCGUUCCUCAUCACGUAGUCGCAACAGUUCAAGAAAUUCAACUCCAGGC

N-5

AGCAGUAGGGGAACUUCUCCUGCUAGAAUGGCUGGCAAUGGCGGUGAUGCUGCUCUUGCUUUGCUGCUGCUUGAC

AGAUUGAACCAGCUUGAGAGCAAAAUGUCUGGUAAAGGCCAACAACAACAAGGCCAAACUGUCACUAAGAAAUCU

N-6

GCUGCUGAGGCUUCUAAGAAGCCUCGGCAAAAACGUACUGCCACUAAAGCAUACAAUGUAACACAAGCUUUCGGC

AGACGUGGUCCAGAACAAACCCAAGGAAAUUUUGGGGACCAGGAACUAAUCAGACAAGGAACUGAUUACAAACAU

N-7 N-8

UGGCCGCAAAUUGCACAAUUUGCCCCCAGCGCUUCAGCGUUCUUCGGAAUGUCGCGCAUUGGCAUGGAAGUCACA

N-9

CCUUCGGGAACGUGGUUGACCUACACAGGUGCCAUCAAAUUGGAUGACAAAGAUCCAAAUUUCAAAGAUCAAGUC

N-10

AUUUUGCUGAAUAAGCAUAUUGACGCAUACAAAACAUUCCCACCAACAGAGCCUAAAAAGGACAAAAAGAAGAAG

GCUGAUGAAACUCAAGCCUUACCGCAGAGACAGAAGAAACAGCAAACUGUGACUCUUCUUCCUGCUGCAGAUUUG

GAUGAUUUCUCCAAACAAUUGCAACAAUCCAUGAGCAGUGCUGACUCAACUCAGGCCUAA

Supplementary Figure 7. Sequences of crRNAs targeting different sites in the SARS-CoV-2 N-gene (Wuhan-Hu-1)

Supplementary Table 1. crRNA sequences used in this study.

Name	Sequence
crRNA1	GGAUUUAGACUACCCCAAAAACGAAGGGGACUAAAACUAGAUUGCUGUUCU ACCAAGUAAUCCAU
crRNA2	GGAUUUAGACUACCCCAAAAACGAAGGGGACUAAAACAAGAUUGCUGUUCU ACCAAGUAAUCCAU
crRNA3	GGAUUUAGACUACCCCAAAAACGAAGGGGACUAAAACUUGAUUGCUGUUCU ACCAAGUAAUCCAU
crRNA4	GGAUUUAGACUACCCCAAAAACGAAGGGGACUAAAACUACAUUGCUGUUCU ACCAAGUAAUCCAU
crRNA5	GGAUUUAGACUACCCCAAAAACGAAGGGGACUAAAACUAGUUUGCUGUUCU ACCAAGUAAUCCAU
crRNA6	GGAUUUAGACUACCCCAAAAACGAAGGGGACUAAAACUAGAAUGCUGUUCU ACCAAGUAAUCCAU
crRNA7	GGAUUUAGACUACCCCAAAAACGAAGGGGACUAAAACUAGAUAGCUGUUCU ACCAAGUAAUCCAU
crRNA8	GGAUUUAGACUACCCCAAAAACGAAGGGGACUAAAACUAGAUACCUGUUCU ACCAAGUAAUCCAU
crRNA9	GGAUUUAGACUACCCCAAAAACGAAGGGGACUAAAACAAGAUUGGUGUUCU ACCAAGUAAUCCAU
crRNA10	GGAUUUAGACUACCCCAAAAACGAAGGGGACUAAAACAAGAUUGCAGUUCU ACCAAGUAAUCCAU
crRNA11	GGAUUUAGACUACCCCAAAAACGAAGGGGACUAAAACAAGAUUGCUCUUCU ACCAAGUAAUCCAU
crRNA-CoV-N-1	GGAUUUAGACUACCCCAAAAACGAAGGGGACUAAAACAAGGUCUUCCUUGC CAUGUUGAGUGAGA
crRNA-CoV-N-2	GGAUUUAGACUACCCCAAAAACGAAGGGGACUAAAACUAGCUCUUCGGUAG UAGCCAAUUUGGUC
crRNA-CoV-N-3	GGAUUUAGACUACCCCAAAAACGAAGGGGACUAAAACGUUGCAACCCAUAU GAUGCCGUCUUUGU
crRNA-CoV-N-4	GGAUUUAGACUACCCCAAAAACGAAGGGGACUAAAACUUGGCAAUGUUGUU CCUUGAGGAAGUUG
crRNA-CoV-N-5	GGAUUUAGACUACCCCAAAAACGAAGGGGACUAAAACCUUGACUGCCGCCU CUGCUCCCUUCUGC
crRNA-CoV-N-6	GGAUUUAGACUACCCCAAAAACGAAGGGGACUAAAACCCUUGUUGUUGUU GGCCUUUACCAGACA
crRNA-CoV-N-7	GGAUUUAGACUACCCCAAAAACGAAGGGGACUAAAACUUUGUUCUGGACCA CGUCUGCCGAAAGC
crRNA-CoV-N-8	GGAUUUAGACUACCCCAAAAACGAAGGGGACUAAAACAAUUUGCGGCCAAU GUUUGUAAUCAGUU
crRNA-CoV-N-9	GGAUUUAGACUACCCCAAAAACGAAGGGGACUAAAACAUUCCGAAGAACGC UGAAGCGCUGGGGG
crRNA-CoV-N-10	GGAUUUAGACUACCCCAAAAACGAAGGGGACUAAAACGGCACCUGUGUAG GUCAACCACGUUCCC

Supplementary Table 2. Target RNA sequences used in this study.

Name	Sequence
tgRNA1	GGGGGCCAGUGAAUUCGAGCUCGGUACCCGGGGAUCCUCUAGAAAUAUGGAUUAC<br>UUGGUAGAACAGCAAUCUACUCGACCUGCAGGCAUGCAAGCUUGGCGUAAUCAUGG<br>UCAUAGCUG
tgRNA2	GGGGGCCAGUGAAUUCGAGCUCGGUACCCGGGGAUCCUCUAGAAAUAUGGAUUAC<br>UUGGUAGAACAGCAAUGUACUCGACCUGCAGGCAUGCAAGCUUGGCGUAAUCAUGG<br>UCAUAGCUG
tgRNA3	GGGGGCCAGUGAAUUCGAGCUCGGUACCCGGGGAUCCUCUAGAAAUAUGGAUUAC<br>UUGGUAGAACAGCUAUGUACUCGACCUGCAGGCAUGCAAGCUUGGCGUAAUCAUG<br>GUCAUAGCUG
SARS-CoV-N	AUGUCUGAUAAUGGACCCCAAAAUCAGCGAAAUGCACCCCGCAUUACGUUUGGUGG<br>ACCCUCAGAUUCAACUGGCAGUAACCAGAAUGGAGAACGCAGUGGGGCGCGAUCAA<br>AACAACGUCGGCCCCAAGGUUUACCCAAUAAUACUGCGUCUUGGUUCACCGCUCUC<br>ACUCAACAUGGCAAGGAAGACCUUAAAUUCCCUCGAGGACAAGGCGUUCCAAUUAA<br>CACCAAUAGCAGUCCAGAUGACCAAAUUGGCUACUACCGAAGAGCUACCAGACGAA<br>UUCGUGGUGGUGACGGUAAAAUGAAAGAUCUCAGUCCAAGAUGGUAUUUCUACUAC<br>CUAGGAACUGGGCCAGAAGCUGGACUUCCCUAUGGUGCUAACAAAGACGGCAUCAU<br>AUGGGUUGCAACUGAGGGAGCCUUGAAUACACCAAAAGAUCACAUUGGCACCCGCA<br>AUCCUGCUAACAAUGCUGCAAUCGUGCUACAACUUCCUCAAGGAACAACAUUGCCA<br>AAAGGCUUCUACGCAGAAGGGAGCAGAGGCGGCAGUCAAGCCUCUUCUCGUUCCU<br>CAUCACGUAGUCGCAACAGUUCAAGAAAUUCAACUCCAGGCAGCAGUAGGGGAACU<br>UCUCCUGCUAGAAUGGCUGGCAAUGGCGGUGAUGCUGCUCUUGCUUUGCUGCUGC<br>UUGACAGAUUGAACCAGCUUGAGAGCAAAAUGUCUGGUAAAGGCCAACAACAACAA<br>GGCCAAACUGUCACUAAGAAAUCUGCUGCUGAGGCUUCUAAGAAGCCUCGGCAAAA<br>ACGUACUGCCACUAAAGCAUACAAUGUAACACAAGCUUUCGGCAGACGUGGUCCAG<br>AACAAACCCAAGGAAAUUUUGGGGACCAGGAACUAAUCAGACAAGGAACUGAUUACA<br>AACAUUGGCCGCAAAUUGCACAAUUUGCCCCCAGCGCUUCAGCGUUCUUCGGAAUG<br>UCGCGCAUUGGCAUGGAAGUCACACCUUCGGGAACGUGGUUGACCUACACAGGUG<br>CCAUCAAAUUGGAUGACAAAGAUCCAAAUUUCAAAGAUCAAGUCAUUUUGCUGAAUA<br>AGCAUAUUGACGCAUACAAAACAUUCCCACCAACAGAGCCUAAAAAGGACAAAAAGA<br>AGAAGGCUGAUGAAACUCAAGCCUUACCGCAGAGACAGAAGAAACAGCAAACUGUG<br>ACUCUUCUUCCUGCUGCAGAUUUGGAUGAUUUCUCCAAACAAUUGCAACAAUCCAU<br>GAGCAGUGCUGACUCAACUCAGGCCUAA

Supplementary Table 3. FQ reporter used in this study.

Name	Sequence
FQ reporter	5′-/56-FAM/rUrUrUrUrU/3IABkFQ/-3′

Supplementary Table 4. Primers used in this study.

Name	Sequence
crRNA-T7P-f	GGATCCTAATACGACTCACTATA
crRNA1-r	ATGGATTACTTGGTAGAACAGCAATCTAGTTTTAGTCCCCTTCGTTTTTGGGGTAGT<br>CTAAATCCTATAGTGAGTCGTATTAGGATCC
crRNA2-r	ATGGATTACTTGGTAGAACAGCAATCTTGTTTTAGTCCCCTTCGTTTTTGGGGTAGT<br>CTAAATCCTATAGTGAGTCGTATTAGGATCC
crRNA3-r	ATGGATTACTTGGTAGAACAGCAATCAAGTTTTAGTCCCCTTCGTTTTTGGGGTAGT<br>CTAAATCCTATAGTGAGTCGTATTAGGATCC
crRNA4-r	ATGGATTACTTGGTAGAACAGCAATGTAGTTTTAGTCCCCTTCGTTTTTGGGGTAGT<br>CTAAATCCTATAGTGAGTCGTATTAGGATCC

crRNA5-r	ATGGATTACTTGGTAGAACAGCAAACTAGTTTTAGTCCCCTTCGTTTTTGGGGTAGT CTAAATCCTATAGTGAGTCGTATTAGGATCC
crRNA6-r	ATGGATTACTTGGTAGAACAGCATTCTAGTTTTAGTCCCCTTCGTTTTTGGGGTAGT CTAAATCCTATAGTGAGTCGTATTAGGATCC
crRNA7-r	ATGGATTACTTGGTAGAACAGCTATCTAGTTTTAGTCCCCTTCGTTTTTGGGGTAGT CTAAATCCTATAGTGAGTCGTATTAGGATCC
crRNA8-r	ATGGATTACTTGGTAGAACAGGAATCTAGTTTTAGTCCCCTTCGTTTTTGGGGTAGT CTAAATCCTATAGTGAGTCGTATTAGGATCC
crRNA9-r	ATGGATTACTTGGTAGAACACCAATCTAGTTTTAGTCCCCTTCGTTTTTGGGGTAGT CTAAATCCTATAGTGAGTCGTATTAGGATCC
crRNA10-r	ATGGATTACTTGGTAGAACTGCAATCTAGTTTTAGTCCCCTTCGTTTTTGGGGTAGT CTAAATCCTATAGTGAGTCGTATTAGGATCC
crRNA11-r	ATGGATTACTTGGTAGAAGAGCAATCTAGTTTTAGTCCCCTTCGTTTTTGGGGTAGT CTAAATCCTATAGTGAGTCGTATTAGGATCC
crRNA-CoV-N-1-r	CCCCCAGCGCTTCAGCGTTCTTCGGAATGTTTTAGTCCCCTTCGTTTTTGGGGTAG TCTAAATCCTATAGTGAGTCGTATTAGGATCC
crRNA-CoV-N-2-r	CAACAGAGCCTAAAAAGGACAAAAAGAAGTTTTAGTCCCCTTCGTTTTTGGGGTAG TCTAAATCCTATAGTGAGTCGTATTAGGATCC
crRNA-CoV-N-3-r	GGGAACGTGGTTGACCTACACAGGTGCCGTTTTAGTCCCCTTCGTTTTTGGGGTA GTCTAAATCCTATAGTGAGTCGTATTAGGATCC
crRNA-CoV-N-4-r	GCTTTCGGCAGACGTGGTCCAGAACAAAGTTTTAGTCCCCTTCGTTTTTGGGGTAG TCTAAATCCTATAGTGAGTCGTATTAGGATCC
crRNA-CoV-N-5-r	CTTCTAAGAAGCCTCGGCAAAAACGTACGTTTTAGTCCCCTTCGTTTTTGGGGTAG TCTAAATCCTATAGTGAGTCGTATTAGGATCC
crRNA-CoV-N-6-r	CCAGGCAGCAGTAGGGGAACTTCTCCTGGTTTTAGTCCCCTTCGTTTTTGGGGTAG TCTAAATCCTATAGTGAGTCGTATTAGGATCC
crRNA-CoV-N-7-r	GCAGAAGGGAGCAGAGGCGGCAGTCAAGGTTTTAGTCCCCTTCGTTTTTGGGGTA GTCTAAATCCTATAGTGAGTCGTATTAGGATCC
crRNA-CoV-N-8-r	TACCTAGGAACTGGGCCAGAAGCTGGACGTTTTAGTCCCCTTCGTTTTTGGGGTAG TCTAAATCCTATAGTGAGTCGTATTAGGATCC
crRNA-CoV-N-9-r	GACCAAATTGGCTACTACCGAAGAGCTAGTTTTAGTCCCCTTCGTTTTTGGGGTAG TCTAAATCCTATAGTGAGTCGTATTAGGATCC
crRNA-CoV-N-10-r	GTGGGGCGCGATCAAAACAACGTCGGCCGTTTTAGTCCCCTTCGTTTTTGGGGTA GTCTAAATCCTATAGTGAGTCGTATTAGGATCC
tgRNA1-r	CAGCTATGACCATGATTACGCCAAGCTTGCATGCCTGCAGGTCGAGTAGATTGCTG TTCTACCAAGTAATCCATATTTCTAGAGGATCCCCGGGTACCGAGCTCGAATTCACT GGCCcccTATAGTGAGTCGTATTAGGATCC
tgRNA2-r	CAGCTATGACCATGATTACGCCAAGCTTGCATGCCTGCAGGTCGAGTACATTGCTG TTCTACCAAGTAATCCATATTTCTAGAGGATCCCCGGGTACCGAGCTCGAATTCACT GGCCcccTATAGTGAGTCGTATTAGGATCC
tgRNA3-r	CAGCTATGACCATGATTACGCCAAGCTTGCATGCCTGCAGGTCGAGTACATAGCTGT TCTACCAAGTAATCCATATTTCTAGAGGATCCCCGGGTACCGAGCTCGAATTCACTG GCCcccTATAGTGAGTCGTATTAGGATCC
CoV-N-FL-PCR-f	GGATCCTAATACGACTCACTATAGGATGTCTGATAATGGACCCCAAAATCAGC
CoV-N-FL-PCR-r	TTAGGCCTGAGTTGAGTCAGCACTG

Supplementary Table 5. Cost /micro-chamber device.

Material	Cost (US$)
32 mm × 24 mm cover glass	0.10
KOH solution	< 0.10
Perfluoro-polymer (CYTOP)	0.30
Photoresist (AZ P4620)	0.10
Developer (AZ300 MIF)	0.80
Acetone	< 0.10
Isopropanol	< 0 .10
Frame chamber seal	1.90
Glass block with an inlet port	2.00
Total	5.20

Supplementary Table 6. Cost/ assay mixture.

Material	Cost (US$)
Buffer D<br>(20 mM HEPES (pH 7.5), 150 mM KCl,<br>10 mM $MgCl_2$, and 0.5 mM DTT)	< 0.10
FQ reporter	3.30
Triton X-100	< 0.10
Alexa Fluor™ 647 C_2 Maleimide	0.60
Total	3.90
