# Supplementary material for: Amplification-free RNA detection with CRISPR–Cas13
Source: Commun Biol. 2021 Apr 19;4:476. doi: 10.1038/s42003-021-02001-8 (PMC8055673; doi:10.1038/s42003-021-02001-8)
Supplement: Supplementary file 2 — Description of Additional Supplementary Files [file 42003_2021_2001_MOESM2_ESM.pdf]

Description of Additional Supplementary Files

File name: Supplementary Data 1

Description: Source data for main figures.
