# Supplementary material for: Amplification-free RNA detection with CRISPR–Cas13
Source: Commun Biol. 2021 Apr 19;4:476. doi: 10.1038/s42003-021-02001-8 (PMC8055673; doi:10.1038/s42003-021-02001-8)
Supplement: Supplementary file 4 — Reporting Summary [file 42003_2021_2001_MOESM4_ESM.pdf]

nature research

Corresponding author(s): Rikiya Watanabe

Last updated by author(s): Mar 8, 2021

# Reporting Summary

Nature Research wishes to improve the reproducibility of the work that we publish. This form provides structure for consistency and transparency in reporting. For further information on Nature Research policies, see our Editorial Policies and the Editorial Policy Checklist.

## Statistics

For all statistical analyses, confirm that the following items are present in the figure legend, table legend, main text, or Methods section.

| n/a | Confirmed | |
|---|---|---|
| ☐ | ☒ | The exact sample size ($n$) for each experimental group/condition, given as a discrete number and unit of measurement |
| ☐ | ☒ | A statement on whether measurements were taken from distinct samples or whether the same sample was measured repeatedly |
| ☒ | ☐ | The statistical test(s) used AND whether they are one- or two-sided<br>*Only common tests should be described solely by name; describe more complex techniques in the Methods section.* |
| ☒ | ☐ | A description of all covariates tested |
| ☒ | ☐ | A description of any assumptions or corrections, such as tests of normality and adjustment for multiple comparisons |
| ☐ | ☒ | A full description of the statistical parameters including central tendency (e.g. means) or other basic estimates (e.g. regression coefficient) AND variation (e.g. standard deviation) or associated estimates of uncertainty (e.g. confidence intervals) |
| ☒ | ☐ | For null hypothesis testing, the test statistic (e.g. $F$, $t$, $r$) with confidence intervals, effect sizes, degrees of freedom and $P$ value noted<br>*Give P values as exact values whenever suitable.* |
| ☒ | ☐ | For Bayesian analysis, information on the choice of priors and Markov chain Monte Carlo settings |
| ☒ | ☐ | For hierarchical and complex designs, identification of the appropriate level for tests and full reporting of outcomes |
| ☒ | ☐ | Estimates of effect sizes (e.g. Cohen's $d$, Pearson's $r$), indicating how they were calculated |

*Our web collection on statistics for biologists contains articles on many of the points above.*

## Software and code

Policy information about availability of computer code

| | |
|---|---|
| Data collection | We used NIS-Elements AR (Version 4.20 and 4.51, NIKON) to operate laser scanning confocal microscope. |
| Data analysis | We used open-source softwares Fiji/ImageJ(Version 1.52n) and Anaconda3 for Python for data analysis. |

For manuscripts utilizing custom algorithms or software that are central to the research but not yet described in published literature, software must be made available to editors and reviewers. We strongly encourage code deposition in a community repository (e.g. GitHub). See the Nature Research guidelines for submitting code & software for further information.

## Data

Policy information about availability of data

All manuscripts must include a data availability statement. This statement should provide the following information, where applicable:

- Accession codes, unique identifiers, or web links for publicly available datasets
- A list of figures that have associated raw data
- A description of any restrictions on data availability

The data that support the findings of this study are available from the corresponding author upon reasonable request.

# Field-specific reporting

# Life sciences study design

All studies must disclose on these points even when the disclosure is negative.

| | |
|---|---|
| Sample size | Sample size calculations were not conducted. We provided the information of sample size of each experiment in each figure legends. We chose the sample sizes to support the conclusions we claimed in the manuscript. |
| Data exclusions | No data was excluded from the analyses. |
| Replication | All experiments performed on the manuscript were successfully replicated more than three times. |
| Randomization | Randomization was not relevant to the study. |
| Blinding | Blinding was not relevant to the study. |

# Reporting for specific materials, systems and methods

We require information from authors about some types of materials, experimental systems and methods used in many studies. Here, indicate whether each material, system or method listed is relevant to your study. If you are not sure if a list item applies to your research, read the appropriate section before selecting a response.

## Materials & experimental systems

| n/a | Involved in the study | |
|---|---|---|
| ☒ | ☐ | Antibodies |
| ☐ | ☒ | Eukaryotic cell lines |
| ☒ | ☐ | Palaeontology and archaeology |
| ☒ | ☐ | Animals and other organisms |
| ☐ | ☒ | Human research participants |
| ☒ | ☐ | Clinical data |
| ☒ | ☐ | Dual use research of concern |

## Methods

| n/a | Involved in the study | |
|---|---|---|
| ☒ | ☐ | ChIP-seq |
| ☒ | ☐ | Flow cytometry |
| ☒ | ☐ | MRI-based neuroimaging |

## Eukaryotic cell lines

Policy information about cell lines

| | |
|---|---|
| Cell line source(s) | VeroE6/TMPRSS2 cells (JCRB 1819). |
| Authentication | The cell line was provided by supplier (JCRB Cell Bank) |
| Mycoplasma contamination | The elimination of mycoplasmas was confirmed by JCRB cell bank. |
| Commonly misidentified lines (See ICLAC register) | No commonly misidentified cell lines were used. |

## Human research participants

Policy information about studies involving human research participants

| | |
|---|---|
| Population characteristics | We enrolled a healthy person who conducted this research, to provide saliva, nasopharyngeal swab, anterior nasal swab, and throat swab samples. |
| Recruitment | A healthy person was recruited from the laboratory member who conducted this research, to obtain saliva, nasopharyngeal swab, anterior nasal swab, and throat swab samples. |
| Ethics oversight | The research protocols were approved by a research ethics committee at RIKEN (Approval number, Wako3 2020-25 (2)). |

Note that full information on the approval of the study protocol must also be provided in the manuscript.
